# Genetic concordance in melanoma: insights from primary tumors and their matched distant metastases

**DOI:** 10.1097/CMR.0000000000001024

**Published:** 2025-02-06

**Authors:** Thamila Kerkour, Ruud W.J. Meijers, Loes M. Hollestein, Anne M.L. Jansen, Ayla Haanappel, Peggy Atmodimedjo, Willeke A.M. Blokx, Bas van Brakel, Tamar E.C. Nijsten, Antien L. Mooyaart

**Affiliations:** a Department of Dermatology; b Department of Pathology, Erasmus MC, Rotterdam; c Department of Pathology, Division of Laboratories, Pharmacy and Biomedical Genetics, UMC Utrecht, Utrecht, the Netherlands

**Keywords:** clonality, distant metastasis, genetic concordance, melanoma, next-generation sequencing

## Abstract

Melanoma metastasis poses a significant challenge due to its aggressive nature and increasing incidence. Confirming the clonal relationship between the primary melanoma and its metastasis is essential to developing reliable prediction models. Here, we compared the genetic profile of primary melanoma and matched metastasis to assess their genetic clonal relationship. Using a targeted sequencing panel encompassing 330 amplicons, we targeted hotspot regions in 41 cancer genes and 154 single nucleotide polymorphisms. The clonal relation between primary and matched metastasis tumors was evaluated by comparing the mutational status and the copy number variations profile in 15 patients with primarily thin melanomas and distant metastases, or with a long latency between the primary melanoma and distant metastasis. Our findings revealed that only about 50% of the analyzed matched primaries and metastases were clonally or likely clonally related, while the remaining sets were either not clonally related or difficult to determine with certainty the clonal relatedness. The findings of our study illustrate the intricate clonal relationships between primary melanoma and metastasis and raise doubts if the metastatic potential is overestimated in the primary tumors. Further investigation with larger cohorts is needed to better understand this complexity of melanoma metastasis and clonality phenomenon, which should be carefully considered when using primary tumor molecular profiles for prognostic model building or therapeutic guidance in metastatic cases.

## Introduction

Melanoma is a highly aggressive malignancy, and its incidence has increased in the past decade [1–3]. The primary stage of melanoma is managed through surgical excision, while more advanced cases can be treated with systemic targeted or immunotherapies [4,5]. Despite the relatively low risk (below 5%) of progression for stage I and II melanoma, the substantial incidence of these melanomas results in a considerable number of patients with initially favorable prognoses developing the metastatic disease [6]. The risk of distant metastasis after stage I melanoma is extremely low (~0.1%) [7]. Therefore, some researchers have suggested that a late distant metastasis after a stage I melanoma may have been a metastasis from another (unknown primary) melanoma but not from the previously diagnosed stage I melanoma [8,9]. In this scenario, accurately determining the clonal origin of metastasis is crucial, especially when those incorrect primary tumors are used for developing new prediction models [10,11]. Genetic concordance analysis is a potent methodology for confirming the matched tumors. However, most studies have analyzed unpaired primary tumors, metastatic tumors, or both, from distinct patients. Furthermore, our recent meta-analysis revealed that the studies that analyzed matched samples mainly focused on common driver mutations in *BRAF* and *NRAS* genes, while other genetic mutations or patterns were not included [12]. This limited approach overlooks other potentially relevant genetic events that could provide a more comprehensive understanding of clonal relationships. Elucidating the clonal mutations and heterogeneous patterns across matched primary and metastatic tumors holds significant promise in unraveling the molecular pathways underlying the metastatic progression of early stage melanoma [13]. Such insights are crucial for prognosticating which patients are predisposed to an increased risk of metastasis [14]. However, the effectiveness of such analyses depends on the accurate matching of primary and metastatic tumors, which is laborious. Our current study analyzed the mutational status and allelic loss in primary melanoma and their corresponding distant metastases within a sample of 15 patients. Our primary objective was to ensure the accurate matching of primary and metastatic tumors. The second goal encompassed identifying shared genetic patterns and critical genes associated with melanoma progression.

## Material and methods

### Patient cohort and cases selection

Patients were retrospectively searched and retrieved in the Erasmus Medical Centre database between 2008 and 2018. Patients with a single primary melanoma who developed distant metastasis or developed distant metastasis after a long time (~10 years) after the first diagnosis of a primary melanoma were eligible. Patients were selected based on the availability of the tumor material. Fifteen patients were identified for this project. In addition to the malignant tissues, pathologically confirmed preexisting nevus associated with the primary tumor in four patients was also collected and analyzed for this study. The ethical committee of the Erasmus MC approved the study as ‘Law Medical Research not applicable’ under study number MEC-2023-0643. The study was conducted according to the Code of Conduct for responsible use of human tissue for health research [15].

### Molecular analysis

#### DNA extraction and quality control

A dermatopathologist (A.L.M.) reviewed all tissue tumor sections and selected tumor-rich regions with an estimated minimum tumor cell percentage of 30% or more. Ten consecutive formalin-fixed paraffin-embedded tissue sections 4 μm thick were deparaffinized and stained with hematoxylin before the microdissection of the tumor area. DNA extraction was performed using the Chelex method. Briefly, tumor tissue sections were transferred into tubes with 5% Chelex 100 Resin (Bio-Rad, Hercules, California, USA) cell lysis solution (Promega, Madison, Wisconsin, USA). DNA was isolated by digestion of proteinase K (Roche, Mannheim, Germany) at 56 °C. Proteinase K was inactivated for 10 min at 95 °C. Then, the samples were centrifuged for 5 min at 14 000 rpm to collect cell debris and Chelex resin. The DNA was collected into new tubes and stored at −20 °C until further use. DNA concentrations were measured using a Qubit 2.0 fluorometer (Thermo Fisher Scientific, Waltham, Massachusetts, USA) using the Qubit dsDNA Quantification Assay Kit, as described by the manufacturer. DNA quality control was measured by PCR and samples with 300 bp were considered as samples with good quality.

#### Target generation sequencing, mutation, and allelic imbalance analysis

Targeted next-generation sequencing (NGS) was applied for the samples with good DNA quality using the Erasmus MC amplicon custom diagnostics panel V5.1 (Supplementary Table 1, Supplemental digital content 1, http://links.lww.com/MR/A421). The NGS steps were followed as described previously [16,17]. Erasmus MC amplicon custom diagnostics panel V5.1 comprised 330 amplicons covering 41 genes, multiple hotspot regions in various cancer-related genes, and 154 single nucleotide polymorphisms (SNPs) in multiple tumor suppressor regions to detect copy number variations (CNVs). The metastatic samples of the patients (2 and 11) were already sequenced previously in the Erasmus Medical Centre for diagnosis purposes, using the Erasmus MC amplicons panel V3 (Supplementary Table 2, Supplemental digital content 1, http://links.lww.com/MR/A421). Thus, we retrieved the bam files from the Erasmus Medical Centre database and used them for this study.

#### Mutation and allelic imbalance analysis:

Sequencing data were aligned to the human reference genome hg19. The mapping and the data analysis were performed with the SeqPilot software (JSI Medical Systems GmbH, Kippenheim, Germany). Two experienced molecular scientists (R.W.J.M. and A.M.L.J.) reviewed and selected the detected variants. Briefly, the sample was considered reliable for analysis if at least 80% of the total amplicons had a coverage depth of more than 50 reads. Nonsynonymous somatic point mutations, insertions, deletions affecting protein amino acid sequences, and splice site alterations were included in the analysis. C>T/G>A transitions were considered formalin artifacts and filtered out, when the coverage was below 20% and the tumor percentage was high (>60%). For *TERT* promoter mutations, we only considered the following mutations (C228T, C250T, and C242_C243delinTT). We excluded all variants in the ESP500si or 1000genomes databases with a minor allele frequency higher than ≥1%.

CNVs were evaluated using SNPitty, which visualizes B-allele frequencies from the NGS sequencing data [18]. Two metastatic samples (from patients 2 and 11) were not eligible for the SNPitty analysis because the targeted NGS was performed using panel V3 that does not cover enough amplicons for a reliable CNVs analysis.

To confirm a CNV, we required that at least two heterozygous SNPs showed an imbalance within the chromosomal arm. The variations at the chromosomal arm level were defined as follows: *allelic imbalance for the whole chromosomal arm*: if several SNPs had one allele, which is present in more copies than the other (gain or loss). As our analysis was based solely on the B-allele frequency plot without the intensity plot, we could only detect allelic imbalances, making it challenging to distinguish definitively between gain and loss. *Loss of heterozygosity (LOH*): if several SNPs within the chromosomal arm had one allele deleted, resulting in homozygosity of the remaining allele. *Homozygous deletion (HD*): If both the alleles of a gene are, absent or deleted. Finally, in cases where excessive noise was present in the data, no clear alterations were reported.

### Clonality analysis

Clonality between matched tumors from the same patient was assessed by two independent evaluators separately (R.W.J.M. and A.M.L.J.) both specializing in melanoma molecular diagnostics. Discussions through regular meetings were made to ensure the reliability of data interpretation. The analysis primarily focused on CNVs, which were given greater weight than oncogenic hotspot mutations, like *BRAF* p.V600E and *TERT* promoter mutations, which are common in melanoma. On the other hand, other more rare oncogenic mutations did play a significant role, unless there was uncertainty if it could be a germline mutation. A variant of unknown significance was only included in the comparison if there is evidence that it is not a germline polymorphism. For example, through its absence in one of the lesions or the nevus.

Since CNVs detection in this study was based on a limited targeted NGS panel, the exact size of a CNV (focal or complete chromosome) could not be determined. We placed more emphasis on partial CNVs of the chromosomal arm due to their higher specificity within a tumor compared to the complete CNV of the chromosomal arm. These regions were analyzed for identical breakpoints, as they provide more reliable evidence of clonal relatedness than broader CNVs. For example, routinely detected CNVs, such as monosomy 9 (often observed in melanocytic lesions), were not considered sufficient evidence of clonal relation unless supported by additional, more specific CNV data. We defined complete CNV of the chromosomal arm if all the SNPs of the chromosomal arm showed imbalance and partial CNV of the chromosomal arm if, less than two SNPs of the chromosomal arm showed imbalance.

The clonality assessment was not exclusively quantitative, as the presence of identical CNVs across entire chromosomes did not automatically confirm clonality. We emphasized the driver mutations in the tumor suppressor genes and the partial CNVs of the same allele. To capture the range of possible clonal relationships, we used the following five categories: (1) Clonally related: the metastasis was derived from the primary tumor, supported by the presence of shared driver mutation in a tumor suppressor gene and the partial CNVs of the same allele. (2) Likely clonally related: clonality was suspected based on shared CNVs, though no definitive evidence was present, and there were no significant discrepancies suggesting otherwise. (3) Not clonally related: strong evidence indicated the matched primary and distant tumors were not derived from the same clone, particularly if strong events (driver mutations in the tumor suppressor genes and the partial CNVs of the same allele) were absent in one sample. (4) Probably not clonally related: low-quality data prevented definitive conclusions but differences in CNVs or mutations raised suspicion of unrelatedness. Finally, (5) No conclusion possible: insufficient or poor-quality data, limited CNVs, and discrepancies between mutations and CNVs made it difficult to determine clonality.

## Results

### Patient and sample characteristics

Nine of the 15 patients were male, and 6 were female. Four patients had tumors with a Breslow thickness of <1 mm, nine with a Breslow thickness of 1–2 mm, and only two had a Breslow thickness of >2 mm. Fourteen primaries were cutaneous melanoma, and one primary was a conjunctival melanoma. The metastatic locations were skin, visceral, and one distant lymph node metastasis. Regarding the time to metastasis, six patients developed metastasis within 5 years, three patients between 5 and 10 years, and six patients after 10 years. Patient demographics and clinical data are summarized in Table 1.

**Table 1:** Clinical characteristics

Patient number	Sex	Age	Primary	BT	Metastasis	Mo
1	F	71	Conjunctiva	0.55	Lung	215
2	M	56	Abdomen	0.81	Colon	71
3	M	70	Orbita	1.1	Liver	47
4	M	37	Back	1.3	Supraclavicular lymph node	178
5	M	56	Trunk	1	Small intestine	119
6	M	75	Shoulder	0.7	Lung	0
7	M	70	Corner of the eye	1.25	Lung	31
8	F	57	Back	0.9	Leg, subcutaneous	48
9	F	42	Trunk	1.65	Leg	204
10	F	59	Trunk	1.3	Vulvar/genital region	84
11	M	67	Arm	1.7	Lung	180
12	F	32	Back	1.9	Leg	48
13	F	74	Arm	1.2	Liver	168
14	M	67	Trunk	8.2	Lung	48
15	M	75	Arm	6	Liver	144

BT, Breslow-thickness (mm); Mo, time in months between the diagnosis of the primary tumor and the distant metastasis.

### Mutation patterns and allelic loss

Among the 41 targeted genes, variants in 11 genes were identified across the 15 patients (Table 2). The most frequently observed variants were *BRAF* gene, found in 19 tumors, with *BRAF* p.V600E detected in eight primary tumors, six metastases, and one nevus. Additionally, the *BRAF* p.V600K variant was noted in three tumors and *BRAF* p.D594N in one sample. The *TERT* promoter hotspot mutations were identified in 18 tumors (11 primaries and 7 metastases). The third most common mutation was in *NRAS* gene, specifically the amino acid position p.Q61, observed in four primary tumors and three metastases. *BRAF* p.V600E and *NRAS* p.Q61 mutations were mutually exclusive within all the samples. The *TP53* gene was mutated in four metastases and three primary tumors, while additional mutations in *CDKN2A*, *MAP2K1*, *SMAD4*, and *MET* genes were detected in one or two samples each.

**Table 2:** Overview of the detected mutations in the analyzed samples

Patient	Primary	Metastasis	Nevus
1	TERT p.C250T (56%) NRAS p.Q61K (33%)	TERT p.C250T (35%) BRAF p.V600K (30%) RET p.E632K (VUS) (48%)	
2	BRAF p.V600E (45%) TERT p.C228T (44%)		
3	BRAF p.V600E (43%) MET p.M1013I (VUS) (30%) TERT p.C250T (20%)	BRAF p.V600E (57%) MET p.M1013I (VUS) (29%) TERT p.C250T (29%)	BRAF p.V600E (40%)
4	BRAF p.V600E (54%) TERT p.C250T (26%)	BRAF p.V600E (55%) PIK3CA p.E545K (20%)	
5	BRAF p.V600E (33%) TERT p.C242_C243delinTT (35%)	BRAF p.V600E (88%) TERT p.C242_C243delinTT (61%)	
6	BRAF p.D594N (24%) MAP2K1 p.P124R (31%) TERT p.C250T (21%)	TERT p.C250T (44%)	No mutations
7	BRAF p.I582M (VUS) (19%) TERT p.C228T(58%)	BRAF p.I582M (VUS) (10%)	No mutations
		TERT p.C228T (51%)	
8	BRAF p.V600K (28%)	BRAF p.V600K (50%)	No mutations
		CDKN2A p.E88* (70%)	
9	CDKN2A p.W110* (73%) BRAF p.V600E (48%)	CDKN2A p.W110* (52%) BRAF p.V600E (53%)	
10	BRAF p.V600E (31%) TERT p.C250T (25%)	BRAF p.V600E (40%) TERT p.C250T (61%)	
11	NRAS p.Q61W (40%) TP53: c.96+1G>A (16%)	NRAS p.Q61W (70%) TP53: c.96+1G>A (74%)	
12	BRAF p.V600E (53%) CDKN2A p.Q80* (56%) TP53 p.R248W (52%) TERT p.C250T (34%)	BRAF p.V600E (50%) CDKN2A p.Q80* (47%) TP53 p.R248W (47%)	
13	NRAS p.Q61R (32%) TERT p.C250T (30%) SMAD4 p.R361C (36%) CDKN2A p.G89V (VUS) (48%) FBXW7 p.L483F (VUS) (28%)	NRAS p.Q61L (60%) TERT p.C250T (56%)	
14	MAP2K1 p.P124S (44%) TERT p.C250T (41%) NRAS p.Q61K (64%) TP53 p.R280K (68%)	NRAS p.Q61K (54%) TP53 p.R280K (81%)	
15	BRAF p.V600E (44%)	BRAF p.V600E (27%) TP53 p.V197E (53%)	

The percentage indicates the variant allele frequency.

VUS, variant of unknown significance.

Fourteen chromosomal regions were analyzed across 17 patients (1p, 3p, 5q, 7p, 8p, 9p, 10q, 11q, 13q, 17p, 17q, 18q, 19p, 19q). All primary lesions and metastases displayed at least one CNV (Table 3). The most common CNVs, LOH of chromosomes arm 9p and 10q, with LOH observed in seven patients. Allelic imbalance was the second most common alteration, found in 8p, 1p, and 7p. LOH or allelic imbalance was also observed in regions such as 3p, 5q, 17p, and 19q, but these alterations were less frequent. Finally, in certain regions, results were inconclusive or not informative due to poor-quality data.

**Table 3:**
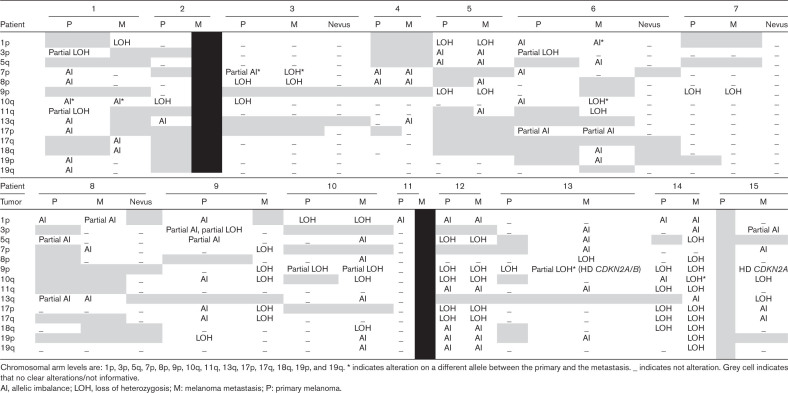
Overview copy number alteration at the chromosomal arm level

### Clonal relationship between primary and distant metastasis tumor

Using CNV data and mutational profiles, we determined the clonal relationship between the matched set of primary tumors and distant metastases (Table 4). For the Patient 1, the primary tumor showed an *NRAS* p.Q61K mutation with multiple CNVs including an allelic imbalance of chromosome 7p, 8p, 13q, and 17p, while the metastasis had a *BRAF* p.V600K mutation and allelic imbalances on different chromosomes. These elements lead to the conclusion that these tumors are not clonally related. For Patient 2, the low quality of the data made it impossible to draw any conclusions. In Patient 3, the primary tumor had different allele that had an allelic imbalance in chromosomal arm 10q than the metastasis, which is a strong argument for them not being clonally related. While the presence of a unique *MET* p.M1013I variant in primary tumor and metastasis, but not in the nevus (making it unlikely to be a polymorphism) supports possible clonal relatedness between the primary and the matched metastasis. Since there is discrepancy between the conclusions based on the mutations and the CNVs data, we could not draw a final conclusion regarding the clonal relatedness between the primary and the matched metastasis. The fact that no mutations were found in the nevus means that no conclusion can be drawn whether or not this nevus was related to the primary tumor.

**Table 4:** Clonal relationship between primary and distant metastasis tumors.

Patient number	Decision regarding the clonality between primary and matched metastasis	Key molecular basis for clonal relatedness decision
1	Not clonally related	Primary: *NRAS* p.Q61K mutation and allelic imbalance of chromosome 7p, 8p, 13q, and 17p, Metastasis: *BRAF* p.V600K mutation and allelic imbalances on different chromosomes
2	No conclusion	Poor quality data
3	No conclusion	Absence of sufficient arguments for either clonality or absence of clonality
4	Probably clonally related	Allelic imbalance on chromosomal arm 13q
5	Clonally related	The overall shared CNVs
6	Probably not clonally related	Absence of *BRAF* p.D594N in the metastasis No other driver mutations detected in the metastasis
7	Probably clonally related	No significant events were identified
8	Probably clonally related	Presence of *BRAF* p.V600K in both the primary and matched metastasis
9	Probably not clonally related	The overall CNVs of primary and metastasis is against the clonal relatedness
10	Probably clonally related	The overall shared CNVs with additional new CNVs acquired in the metastasis
11	Clonally related	Presence of *NRAS* p.Q61W in both the primary and metastasis
12	Clonally related	Shared CNVs and mutation profile of the primary and metastasis
13	Not clonally related	Different mutation in *NRAS* position p.Q61 was found in primary and metastasis.
14	Clonally related	Presence of *NRAS* p.Q61K in both the primary and distant metastasis
15	No conclusion	Poor quality data

In Patient 4, allelic imbalance on chromosomal arm 13q in the metastasis suggests that the primary tumor and metastasis are probably related, though no strong mutational events were observed to confirm this definitively. For Patient 5, several shared CNVs events support the conclusion that the primary tumor and metastasis are clonally related. In Patient 6, a class III *BRAF* p.D594N (kinase-impaired) with additional *MAP2K1* activating mutation (requiring RAS activation) was detected in the primary tumor but not in the metastasis. Additionally, several CNVs differed between the primary tumor and the metastasis, which also points toward the absence of a clonal relationship. Since no clear driver mutation was identified in the metastasis, we cannot draw a definitive conclusion. In theory, a common driver mutation could still be present that was not detected with our NGS technique. As conclusion, the available data suggest a lack of clonal relatedness. The fact that these driver mutations were not found in the nevus is difficult to comprehend, other than that this lesion might also not be related to the primary tumor. For Patient 7, no significant events were identified to clearly differentiate between the primary tumor and metastasis, leading to the conclusion that the primary and metastasis tumors are probably clonally related. However, no conclusions could be drawn regarding the nevus. In Patient 8, the *BRAF* p.V600K mutation was present in both the primary and metastatic tumors, but absent in the nevus, indicating that the nevus is not clonally related to either. While *BRAF* p.V600K is found in only 3% of diagnostic cases, and thus can possibly be indicative of a clonal relationship. Differences in CNVs profile between the primary and metastasis prevented a definitive conclusion regarding clonality. For Patient 9, the primary tumor displayed LOH in regions 3p and 19q, which were absent in the metastasis. Conversely, the metastasis exhibited LOH in regions 9p and 10q, which were not present in the primary tumor. Additionally, *CDKN2A* p.W110* was found in bot needs primary and distant metastasis, while it needs to be confirmed in normal cells to exclude germline mutation. Therefore, CNVs suggest that the primary tumor and metastasis are probably not clonally related.

In Patient 10, no clear events were observed to confirm the clonality, while additional variants were detected in the metastasis, leading to the conclusion that the primary and metastatic tumors are probably clonally related. In Patient 11, CNVs data were lacking for the metastasis, but the presence of the *NRAS* p.Q61W mutation in both the primary and the metastasis provided enough evidence to confirm clonality, as this is a rare *NRAS* variant on codon 61.

For Patient 12, both CNVs and mutational analysis strongly support clonal relatedness between the primary tumor and metastasis. For Patient 13, the different *NRAS* mutations in the primary and metastatic tumors suggest that the matched primary and metastasis are not clonally related. In Patient 14, the presence of the *NRAS* p.Q61K mutation in both the primary and metastatic tumors indicates they are clonally related. Finally, for Patient 15, poor-quality data prevented any conclusions from being drawn regarding clonality.

## Discussion

In this study, we explored the genetic concordance between primary melanomas and their presumed matched distant metastases by investigating CNVs and mutations in 41 cancer-related genes. We analyzed 15 patients, each with primary melanoma and matched metastatic tumors, and included nevus material in four cases. Out of the 15 patients, the primary and metastatic tumors were definitely clonally related in four patients and likely clonally related in five others. In four patients, there was no evidence to suggest clonality between primary and metastatic sets, and for the rest of the three patients, poor-quality data, or lacking of enough stranger arguments prevented any definitive conclusion. Our study indicates that clonal relatedness is not always straightforward.

In our previous meta-analysis, we showed that most studies focused on oncogenic point mutations to identify the clonal origin between primary cutaneous melanoma and their associated metastasis [12]. We observed a concordance rate of approximately 90% for *BRAF* status between primary tumors and metastases. Although it is unclear whether the observed discrepancies stem from differences in clonal origin or technical artifacts, this finding suggests that clear clonal relationships may not always be evident in certain cases. Here, when we compared the tumors, based on multiple CNVs and mutations, we showed that in several cases, the primary tumor exhibited a genetic profile that is distinct from its presumed corresponding metastasis. An explanation could be that tumor cell dissemination to distant sites may involve more complex mechanisms than previously thought. The new recent study by Bezrookove *et al.* reported also inconsistent patterns of changes in variants allele frequency when comparing the primary melanoma to their matched lymph node and distant metastasis [19]. The study indicates the patterns of cancer cells with copy number alteration changes during the metastasis cascade of melanoma.

The intra-tumor molecular heterogeneity has been already proved in several studies [20,21]. We know that not all the cells within the primary tumor have the metastasis capacity, therefore, one possible explanation for this genetic discordance in our patients, is the polyclonal origin. In this scenario, a small cell fraction considered as a minor clone present in the primary tumor could give rise to the metastasis [22]. The minor clone might be undetectable with the current NGS technique due to its low frequency, leading to different genetic profiles in the primary and matched metastasis. The second possibility for this discrepancy is that the primary melanoma sample analyzed may not be the true primary origin of the metastasis, as found in Patient 1, where the primary and metastatic tumors exhibited different genetic patterns and time in months between diagnosis primary and metastasis was notably long (215 months). Therefore, we rise concerns about the reliability of using primary tumor data to guide treatment decisions for metastases, especially when metastasis biopsy material is not available. This concern extends to the use of the primary tumor molecular profiles to build the prognostic models. If the genetic differences between primary and metastatic tumors is present, relying only on primary tumor data could affect prediction accuracy. Understanding the clonality relatedness before developing the model would help ensure more reliable results, and incorporating matched metastatic data, if available, may further strengthen the model.

Among the detected alterations, the allelic imbalance and the LOH particularly in on chromosomal arms 9p and 10q were observed in both the primary and the metastatic tumors. These variations may play a role in melanoma metastasis as suggested previously [23]. The mutations together with allelic copies should be investigated with more comprehensive profiles to understand the metastasis progression. Despite the advancements in genetic analysis techniques, determining clonality remains challenging due to the natural heterogeneity in melanoma. Inconclusive results in two patients were due to poor-quality data or lack of significant alteration events, illustrate the limitations of the current methodologies, and the need for more sensitive techniques that can generate higher-quality data to trace the origins and evolution of metastatic tumors more accurately.

In one case of our study (Patient 3), shared genetic alterations were detected between primary tumor, distant metastasis, and the nevi, suggesting that nevi may act as precursors for both the primary and the distant metastasis tumors. This observation highlights the potential importance of benign lesions in melanoma progression and metastasis. We also showed that CNVs were not detected in nevi, confirming that those indeed are associated with tumor progression [24].

The strengths of our study lie in its comprehensive approach to genomic analysis, which included the evaluation of multiple cancer-related genes and CNVs using a targeted NGS panel.

Nevertheless, our study has limitations. The small sample size and retrospective patient selection limit our ability to generalize the findings or draw strong correlations with clinicopathological characteristics. Moreover, our targeted gene panel, while informative, covered only a subset of cancer driver genes, with roughly 20% of the included genes specifically implicated in melanoma. Expanding the panel to include a broader range of melanoma-specific genes would enhance our capacity to capture the full spectrum of genomic differences between primary and metastatic tumors; moreover, including normal tissue analysis would allow us to exclude germline mutations, providing greater precision in identifying tumor-specific alterations. Broader CNV analysis, such as through SNP array analysis, can strengthen the reliability of the conclusions drawn and, in some cases, eliminate any doubt. We attempted to conduct SNP array analysis on our material, but the quality of the DNA was insufficient due to the age of the samples.

In conclusion, our study offers evidence for both clonal relatedness and independent evolution between primary tumors and distant metastases. These findings emphasize the complexity of melanoma metastasis. Future studies with larger sample sizes and more comprehensive genomic analyses are essential to better understand the clonal relationships between primary melanomas and their distant metastases and unravel the intricate dynamics of tumor progression and metastatic dissemination.

## Acknowledgements

A.L.M., W.A.M.B., B.B., and A.M.L.J. contributed in the conceptualization. T.K., R.W.J.M., A.M.L.J., P.A., and A.H. contributed in the methodology. R.W.J.M., T.K., A.M.L.J., and P.A. contributed in the software. A.M.L.J., R.W.J.M., and A.L.M. contributed in the data interpretation. A.L.M. and A.H. contributed in the resources. T.K. contributed in the writing – original draft preparation. T.K., R.W.J.M., L.H., T.E.C.N., W.A.M.B., and A.L.M. contributed in the writing – review and editing. T.K. contributed in the visualization. A.M. and L.H. contributed in the supervision. All authors have read and agreed to the published version of the manuscript.

### Conflicts of interest

There are no conflicts of interest.

## Supplementary Material



## References

[1] Bucchi L Mancini S Zamagni F Crocetti E Dal Maso L Ferretti S. Patient presentation, skin biopsy utilization and cutaneous malignant melanoma incidence and mortality in northern Italy: trends and correlations. J Eur Acad Dermatol Venereol. 2023; 37:293–302. 36181283 10.1111/jdv.18635 PMC10092783

[2] Dzwierzynski WW. Melanoma risk factors and prevention. Clin Plast Surg. 2021; 48:543–550. 34503715 10.1016/j.cps.2021.05.001

[3] Whiteman DC Olsen CM MacGregor S Law MH Thompson B Dusingize JC. The effect of screening on melanoma incidence and biopsy rates. Br J Dermatol. 2022; 187:515–522. 35531668 10.1111/bjd.21649 PMC9796145

[4] Garbe C Amaral T Peris K Hauschild A Arenberger P Bastholt L. European consensus-based interdisciplinary guideline for melanoma. Part 2: treatment–update 2019. Eur J Cancer. 2020; 126:159–177. 31866016 10.1016/j.ejca.2019.11.015

[5] Schadendorf D Fisher DE Garbe C Gershenwald JE Grob J-J Halpern A. Melanoma. Nat Rev Dis Primers. 2015; 1:1–20. 10.1038/nrdp.2015.3 27188223

[6] Ertekin SS Podlipnik S Riquelme-Mc Loughlin C Barreiro-Capurro A Arance A Carrera C. Initial stage of cutaneous primary melanoma plays a key role in the pattern and timing of disease recurrence. Acta Derm Venereol. 2021; 101:adv00502–adv00502. 34003298 10.2340/00015555-3832 PMC9413807

[7] Bachaud JM Shubinski R Boussin G Chevreau C David JM Viraben R. Stage I cutaneous malignant melanoma: risk factors of loco-regional recurrence after wide local excision and clinical perspectives. Eur J Surg Oncol. 1992; 18:442–448. 1426294

[8] Egberts F Bergner I Krüger S Haag J Behrens HM Hauschild A Röcken C. Metastatic melanoma of unknown primary resembles the genotype of cutaneous melanomas. Ann Oncol. 2014; 25:246–250. 24276025 10.1093/annonc/mdt411

[9] Helvind NM Brinch-Møller Weitemeyer M Chakera AH Hendel HW Ellebæk E Svane IM. Stage-specific risk of recurrence and death from melanoma in Denmark, 2008–2021: a national observational cohort study of 25 720 patients with stage IA to IV Melanoma. JAMA Dermatol. 2023; 159:1213–1222. 37650576 10.1001/jamadermatol.2023.3256 PMC10472263

[10] Ostrovnaya I Olshen AB Seshan VE Orlow I Albertson DG Begg CB. A metastasis or a second independent cancer? Evaluating the clonal origin of tumors using array copy number data. Stat Med. 2010; 29:1608–1621. 20205270 10.1002/sim.3866 PMC3145177

[11] Bradford PT Freedman DM Goldstein AM Tucker MA. Increased risk of second primary cancers after a diagnosis of melanoma. Arch Dermatol. 2010; 146:265–272. 20231496 10.1001/archdermatol.2010.2 PMC3076705

[12] Kerkour T Zhou C Hollestein L Mooyaart A. Genetic concordance in primary cutaneous melanoma and matched metastasis: a systematic review and meta-analysis. Int J Mol Sci. 2023; 24:16281. 38003476 10.3390/ijms242216281 PMC10671327

[13] Braeuer RR Watson IR Wu C-J Mobley AK Kamiya T Shoshan E Bar-Eli M. Why is melanoma so metastatic? Pigment Cell Melanoma Res. 2014; 27:19–36. 24106873 10.1111/pcmr.12172

[14] Richetta AG Valentini V Marraffa F Paolino G Rizzolo P Silvestri V. Metastases risk in thin cutaneous melanoma: prognostic value of clinical-pathologic characteristics and mutation profile. Oncotarget. 2018; 9:32173–32181. 30181807 10.18632/oncotarget.25864 PMC6114949

[15] Commissie Regelgeving Onderzoek. Code of conduct for health research. COREON Foundation; 2022.

[16] Pruis MA Geurts-Giele WRR von der TJH Meijssen IC Dinjens WNM Aerts JGJV. Highly accurate DNA-based detection and treatment results of MET exon 14 skipping mutations in lung cancer. Lung Cancer. 2020; 140:46–54. 31862577 10.1016/j.lungcan.2019.11.010

[17] van Doeveren T Nakauma-Gonzalez JA Mason AS van Leenders GJLH Zuiverloon TCM Zwarthoff EC. The clonal relation of primary upper urinary tract urothelial carcinoma and paired urothelial carcinoma of the bladder. Int J Cancer. 2021; 148:981–987. 33006377 10.1002/ijc.33327 PMC7821318

[18] van Riet J Krol NMG Atmodimedjo PN Brosens E van IJcken WFJ Jansen MPHM. SNPitty: an intuitive web application for interactive B-allele frequency and copy number visualization of next-generation sequencing data. J Mol Diagn. 2018; 20:166–176. 29305224 10.1016/j.jmoldx.2017.11.011

[19] Bezrookove V Kianian S McGeever L Jones R Caressi C Nosrati M. The molecular evolution of melanoma distant metastases. J Investig Dermatol. 2024; 144:2530–2540.e1. 38582370 10.1016/j.jid.2024.03.029

[20] Grzywa TM Paskal W Włodarski PK. Intratumor and intertumor heterogeneity in melanoma. Transl Oncol. 2017; 10:956–975. 29078205 10.1016/j.tranon.2017.09.007 PMC5671412

[21] Mejbel HA Arudra SKC Pradhan D Torres-Cabala CA Nagarajan P Tetzlaff MT. Immunohistochemical and molecular features of melanomas exhibiting intratumor and intertumor histomorphologic heterogeneity. Cancers (Basel). 2019; 11:1714. 31684113 10.3390/cancers11111714 PMC6896082

[22] Janiszewska M Tabassum DP Castaño Z Cristea S Yamamoto KN Kingston NL. Subclonal cooperation drives metastasis by modulating local and systemic immune microenvironments. Nat Cell Biol. 2019; 21:879–888. 31263265 10.1038/s41556-019-0346-x PMC6609451

[23] Conway C Beswick S Elliott F Chang Y-M Randerson-Moor J Harland M. Deletion at chromosome arm 9p in relation to BRAF/NRAS mutations and prognostic significance for primary melanoma. Genes Chromosomes Cancer. 2010; 49:425–438. 20140953 10.1002/gcc.20753 PMC2948432

[24] Ebbelaar CF Jansen AML Bloem LT Blokx WAM. Genome-wide copy number variations as molecular diagnostic tool for cutaneous intermediate melanocytic lesions: a systematic review and individual patient data meta-analysis. Virchows Arch. 2021; 479:773–783. 33851238 10.1007/s00428-021-03095-5 PMC8516778

