# Supplementary material for: Genetic concordance in melanoma: insights from primary tumors and their matched distant metastases
Source: Melanoma Res. 2025 Feb 6;35(3):162–9. doi: 10.1097/CMR.0000000000001024 (PMC12043267; doi:10.1097/CMR.0000000000001024)
Supplement: Supplementary file 1 [file mr-35-162-s001.pdf]

Genetic Concordance in Melanoma: Insights from Primary Tumors and their matched Distant Metastases.

Thamila Kerkour[1], Ruud W. J. Meijers[2], Loes .M Hollestein[1], Anne M. L. Jansen[3], Ayla Haanappel[2], Peggy Atmodimedjo[2], Willeke A. M. Blokx[3], Bas van Brakel[2], Tamar E. C. Nijsten[1], Antien L. Mooyaart [2*]

[1]Department of Dermatology, Erasmus MC Rotterdam, the Netherlands.

[2]Department of Pathology, Erasmus MC Rotterdam, the Netherlands.

[3]Department of Pathology, Division of Laboratories, Pharmacy and Biomedical Genetics, UMC Utrecht, the Netherlands.

* Corresponding author

Antien Mooyaart

Department of Pathology

Dr. Molewaterplein 40

3015GD Rotterdam

Supplementary data:

Supplementary Table 1: Genes included in the next-generation DNA targeted sequencing panel.

Note: Diagnostic V5.1 next-generation sequencing panel. Erasmus Medical Center, Rotterdam.

**Whole gene coverage (open reading frame)**	**Gene**	
	CDKN2A	
	PTEN	
	TP53	
**Hotspot coverage**	**Gene**	**Exons or codons covered**
	AKT1	exon 3
	ALK	exon 20, 22-25
	Amel_X	not applicable
	Amel_Y	not applicable
	APC	exon 16
	ARAF	exon 7
	BRAF	exon 11, 15
	CTNNB1	exon 3, 7, 8
	EGFR	exon 18-21
	ERBB2	exon 19-21
	EZH2	exon 16
	FBXW7	exon 9, 10
	FGFR1	exon 4, 7, 12
	FGFR2	exon 7, 9, 12
	FGFR3	exon 7, 9
	FOXL2	exon 1
	GNA11	exon 4, 5
	GNAQ	exon 4, 5
	GNAS	exon 8, 9

	HRAS	exon 2-4
	IDH1	exon 4
	IDH2	exon 4
	KIT	exon 8, 9, 11, 13, 14, 17
	KRAS	exon 2-4
	MAP2K1	exon 2, 3
	MET	exon 2, 14, 19
	MYD88	exon 5
	NOTCH1	exon 26, 27
	NRAS	exon 2-4
	PDGFRa	exon 12, 14, 18
	PIK3CA	exon 10, 21
	POLD1	exon 12
	POLE	exon 9, 13
	RAF1	exon 7
	RET	exon 11, 16
	RNF43	exon 3, 4, 9
	ROS1	exon 38, 41
	SMAD4	exon 3, 9, 12
	STK11	exon 4, 8
	TERT promotor	promotor region
**Single nucleotide polymorphisms (SNPs)**	**Gene or region**	**Number of SNPs included**
	1p	11 SNPs
	8p	9 SNPs
	chr7	9 SNPs
	19q	9 SNPs
	APC	9 SNPs
	ARID1A	8 SNPs
	ATM	9 SNPs
	BRCA1	9 SNPs
	BRCA2	9 SNPs
	CDKN2A	9 SNPs

	FHIT	9 SNPs
	PTEN	9 SNPs
	RB1	9 SNPs
	SMAD4	9 SNPs
	STK11	9 SNPs
	TP53	9 SNPs
	VHL	9 SNPs
**Total number of amplicons per panel**	330	

Supplementary Table 2: Genes included in the next-generation DNA targeted sequencing panel for the metastatic sample of the patient 10 and 11.

Note: Diagnostic V3 next-generation sequencing panel. Erasmus Medical Center, Rotterdam.

**Whole gene coverage (open reading frame)**	**Gene**	
	TP53	
**Hotspot coverage**	**Gene**	**Exons or codons covered**
	AKT1	codon 17
	ALK	exon 20, 22, 25, codon 1174, 1245
	ARAF	codon 214
	BRAF	codon 469, 600
	CTNNB1	exon 3
	EGFR	exon 18-21
	ERBB2	exon 19-21
	FOXL2	exon 3
	GNA11	codon 209
	GNAQ	codon 209
	GNAS	codon 201, 227
	HRAS	codon 12/13, 61, 117-146
	IDH1	codon 132
	IDH2	codon 140-172
	KIT	exon 8, 9, 11, 13, 17
	KRAS	codon 12/13, 61, 117-146
	MET	codon 375, 1010, 1248
	NRAS	codon 12/13, 61, 117-146
	PDGFRa	exon 12, 14, 18

	PIK3CA	codon 538, 1020
	POLD1	codon 478
	POLE	codon 286, 297, 411, 424
	RAF1	codon 257
**Single nucleotide polymorphisms (SNPs)**	**Gene or region**	**Number of SNPs included**
	1p36	8 SNPs
	TP53	9 SNPs
**Total number of amplicons per panel**		101
